# The Identification of Structural Changes in the Lithium Hexamethyldisilazide–Toluene System via Ultrasonic Relaxation Spectroscopy and Theoretical Calculations

**DOI:** 10.3390/molecules29040813

**Published:** 2024-02-09

**Authors:** Constantine Kouderis, Afrodite Tryfon, Themistoklis A. Kabanos, Angelos G. Kalampounias

**Affiliations:** 1 Physical Chemistry Laboratory, Department of Chemistry, University of Ioannina, GR-45110 Ioannina, Greece; 2 Section of Inorganic and Analytical Chemistry, Department of Chemistry, University of Ioannina, GR-45110 Ioannina, Greece; tkampano@uoi.gr; 3 Institute of Materials Science and Computing, University Research Center of Ioannina (URCI), GR-45110 Ioannina, Greece

**Keywords:** lithium hexamethyldisilazide, ultrasonic relaxation spectroscopy, DFT calculations, molecular docking, self-aggregation, hetero-aggregation

## Abstract

Ultrasonic absorption measurements were carried out over a wide concentration and temperature range by means of a pulse technique to examine the structural mechanisms and the dynamical properties in **li**thium **h**exa**m**ethyl**d**i**s**ilazide (LiHMDS)–toluene solutions. Acoustic spectra revealed two distinct Debye-type relaxational absorptions attributed to the formation of trimers from dimeric and monomer units and to the formation of aggregates between a LiHMDS dimer and one toluene molecule in low and high frequencies, respectively. The formation of aggregates was clarified by means of molecular docking and DFT methodologies. The aggregation number, the rate constants and the thermodynamic properties of these structural changes were determined by analyzing in detail the concentration-dependent relaxation parameters. The low-frequency relaxation mechanism dominates the acoustic spectra in the high LiHMDS mole fractions, while the high-frequency relaxation influences the spectra in the low LiHMDS mole fractions. In the intermediate mole fraction region (0.25 to 0.46), both relaxations prevail in the spectra. The adiabatic compressibility, the excess adiabatic compressibility and the theoretically estimated mean free length revealed a crossover in the 0.25 to 0.46 LiHMDS mole fractions that signified the transition from one structural mechanism related with the hetero-association of LiHMDS dimers with toluene molecules to the other structural mechanism assigned to the formation of LiHMDS trimers. The combined use of acoustic spectroscopy with theoretical calculations permitted us to disentangle the underlying structural mechanisms and evaluate the volume changes associated with each reaction. The results were compared with the corresponding theoretically predicted volume changes and discussed in the context of the concentration effect on intermolecular bonding.

## 1. Introduction

Lithium bis(trimethylsilyl)amide or lithium hexamethyldisilazide (LiHMDS) has attracted research interest in the field of organometallic chemistry due to its eminent properties, such as the formation of various acetylide [1] and lithium enolate [2] organolithium compounds. Aside from its use as a strong non-nucleophilic base in organic chemistry on both the laboratory and industrial scales, as a ligand, it reacts with a wide range of metal halides to form metal bis(trimethylsilyl)amides by the so-called salt metathesis reaction. Due to this ligand, the latter complexes exhibit lipophilicity and thus are soluble in a wide range of non-polar organic solvents [3]. From a structural point of view, LiHMDS forms self- and hetero-aggregates upon dissolution depending on the coordinating or non-coordinating properties of the solvent, a characteristic that is common to other organolithium reagents [3].

When LiHMDS is dissolved in ethers or amines, which are coordinating solvents, then the structure is dominated by monomer and dimer units with one and two solvent molecules binding to lithium centers, respectively [4]. Higher complex oligomers, such as trimers, are formed when aromatics or pentane are used, which are non-coordinating solvents [5]. A trisolvated monomer is formed when ammonia is used as a donor base. This species is stabilized by intermolecular hydrogen bonds [6]. On the other hand, in the solid state, only the trimeric structure is stable [7]. 

Despite the relatively high solubility of LiHMDS and its chemical stability and commercial availability, only a few experimental and computational studies have been focused on the physicochemical properties of LiHMDS in solution state. We report herein a combined spectroscopic and computational study of the aggregation and solvation of LiHMDS in toluene in an effort to elucidate the effect of solution concentration on the structure of these solutions. More specifically, the formation of aggregated species was refined by performing concentration- and temperature-dependent ultrasonic relaxation spectroscopic measurements. Molecular docking and Density Functional Theory (DFT) calculations were also conducted under ambient temperature and pressure conditions that allowed us to disentangle the underlying relaxation processes detected in the acoustic spectra and to improve our comprehensive understanding of the structure and dynamics of LiHMDS solutions.

## 2. Results and Discussion

### 2.1. Structural Processes in LiHMDS Solutions

**Figure 1:**
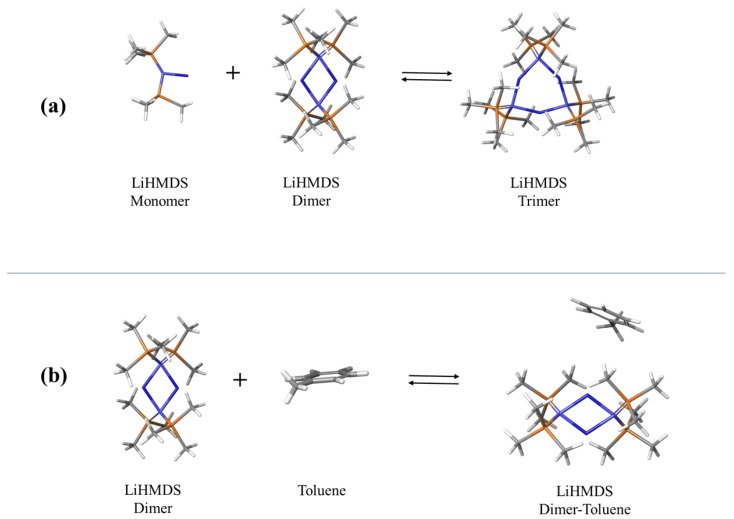
Possible mechanisms proposed for the structure of the LiHMDS organosilicon compound when dissolved in toluene. Schematic representation of the formation of LiHMDS trimer (**a**,**b**) of toluene-complexed LiHMDS dimer. The formation of trimers from monomers and dimers results in a change in Gibbs free energy of −6.97 kcal/mol, whereas the aggregation of dimers and toluene results in a change in Gibbs free energy of 8.91 kcal/mol.

Several mechanisms have been proposed for the structure of the LiHMDS organosilicon compound when dissolved in toluene [4,5,6,8]. In Figure 1a, the formation of trimers from monomer and dimer units is presented. All structures were optimized under tight optimization convergence criteria. The molecular structure of toluene was received from the PubChem electronic database as an SDF file and was also optimized. DFT calculations reported in the literature [8] revealed that the highest degree of aggregation at 298 K is four. However, in non-polar solvents, such as toluene, the formation of dimers and trimers is thermodynamically favorable, with tetramers simply serving as intermediates. The formation of pentamer units has never been reported in the literature to our knowledge.

The high solubility of LiHMDS in toluene indicated an explicit π complexation, resulting in the formation of a toluene-complexed LiHMDS dimer, such as that illustrated in Figure 1b. The aggregate was obtained by means of the AutoDock software (version 4), using the optimized geometries of the LiHMDS dimer and toluene molecules as input, after minimizing energy with respect to the coordinates of atoms and no restrictions on the symmetry. The two reactions can be described as follows:

(1) $$\left[LiHMDS\right]+{\left[LiHMDS\right]}_{2}⇌\;{\left[LiHMDS\right]}_{3}$$

(2) $${\left[LiHMDS\right]}_{2}+Toluene⇌Toluene\cdot {\left[LiHMDS\right]}_{2}$$

The molecular interaction study revealed that the specific pose of the aggregate presented in Figure 1b is the most stable and corresponds to a docking score of −2.91 kcal/mol. The lowest and the highest distances between LiHMDS dimer and toluene molecules that constitute the mixed aggregate were estimated to be theoretically equal to 2.35 Å and 3.48 Å, respectively. The lowest and the highest distances between the Li atom and the toluene ring were 5.05 and 5.47 Å, respectively. The distance between the Li atom and the center of the toluene ring was found to be 5.08 Å. These distances are indeed indicative of metal–pi interaction. The bond distances and angles are presented in the Supplementary Materials. The molecular volumes of all species presented in Figure 1 were estimated theoretically following a specific methodology. For each atom of the molecule, the volume was evaluated from the corresponding wavefunction which was determined by a quantum mechanical calculation. This volume is related to the space included in a contour of a particular electron density of 0.001 electrons/Bohr^3^. Subsequently, the as-obtained wavefunction was integrated to obtain the atomic volume. The sum of all individual atomic volumes specifies the molecular volume. The results are summarized in Table 1.

### 2.2. Ultrasonic Sensing of the Relaxation Processes—Concentration Dependence

**Figure 2:**
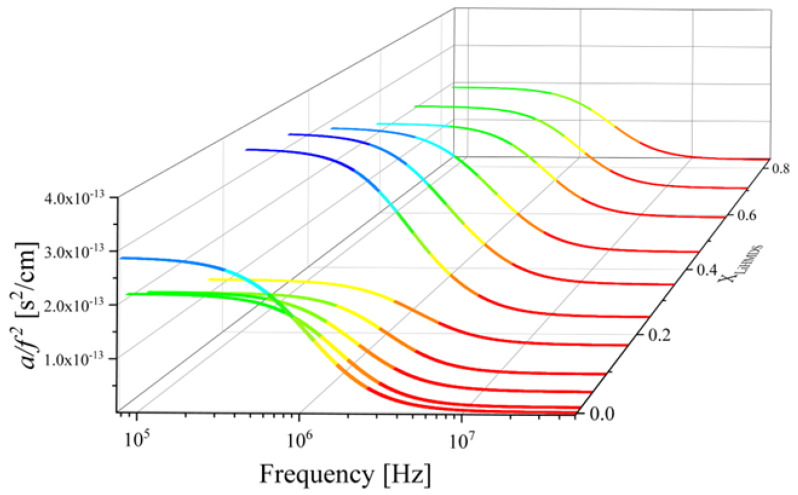
Ultrasound absorption spectra $\left(a/f^{2}\right)$ as a function of frequency for all LiHMDS–toluene solutions at 20 °C. Lines represent the total fitting relaxation profiles for each LiHMDS mole fraction.

In Figure 2, the ultrasonic absorption spectra for the solutions of LiHMDS in toluene recorded at 20 °C are shown. The frequency-reduced absorption coefficients $a/f^{2}$ as a function of frequency *f* were adequately fitted to Debye-type profiles, which is given as follows [9,10]:

(3) $$\frac{a}{f^{2}}=\sum_{i}\frac{A_{i}}{\left[1+{\left(\frac{f}{f_{r,i}}\right)}^{2}\right]}+B$$

where $A_{i}$ is the amplitude of the relaxation of the *i*-th process and *B* is the classical contribution to $a/f^{2}$ due to viscous and thermal losses. The classical contribution to the absorption coefficient is independent from the solution concentration. The relaxation frequency of the *i*-th process is denoted as $f_{r,i}$. The solid lines representing the fitting curves seem to follow a non-monotonous trend with solution concentration. 

**Figure 3:**
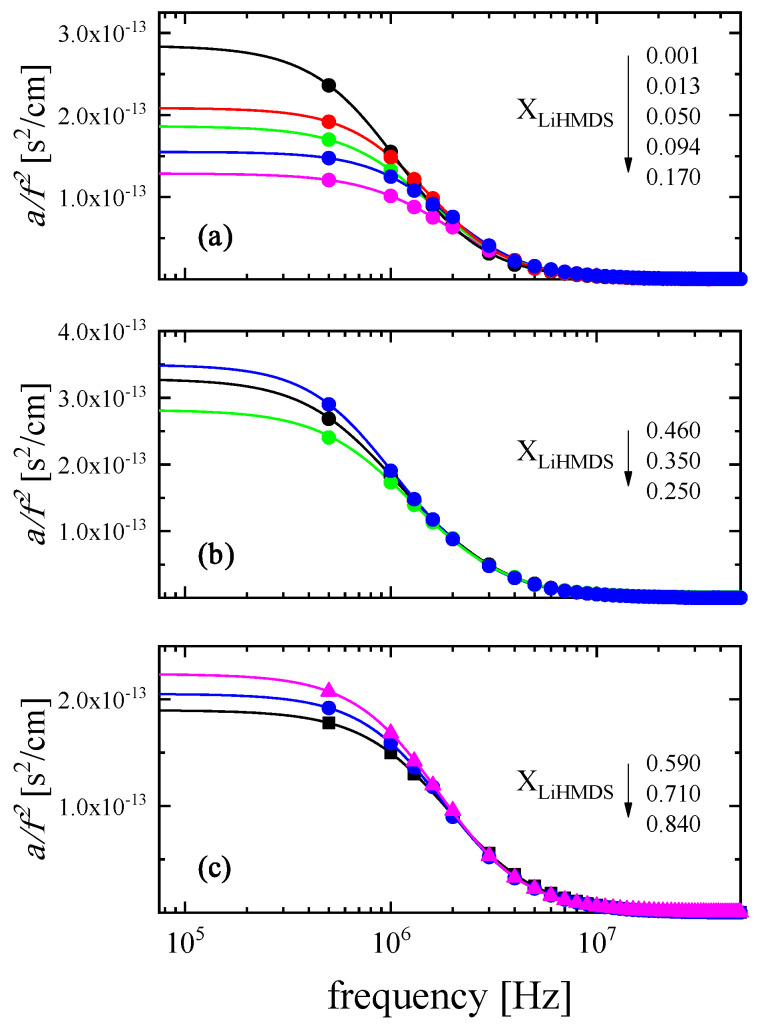
Experimental ultrasound absorption measurements in the frequency reduced form $\left(a/f^{2}\right)$ as a function of frequency in the (**a**) low-, (**b**) intermediate- and (**c**) high-concentration region of LiHMDS–toluene solutions at 20 °C. Lines represent the total fitting relaxation profiles for each solution.

In an effort to quantitatively follow this behavior, we focused our analysis on the acoustic spectra in three different domains, namely the low-, the medium- and the high-mole fraction region of LiHMDS, and the results are illustrated in Figure 3. Lines represent the total fitting relaxation profiles for each solution and the symbols denote the experimental data. From the goodness of fit, it is obvious that the Debye equation is adequate to fit the experimental points.

**Figure 4:**
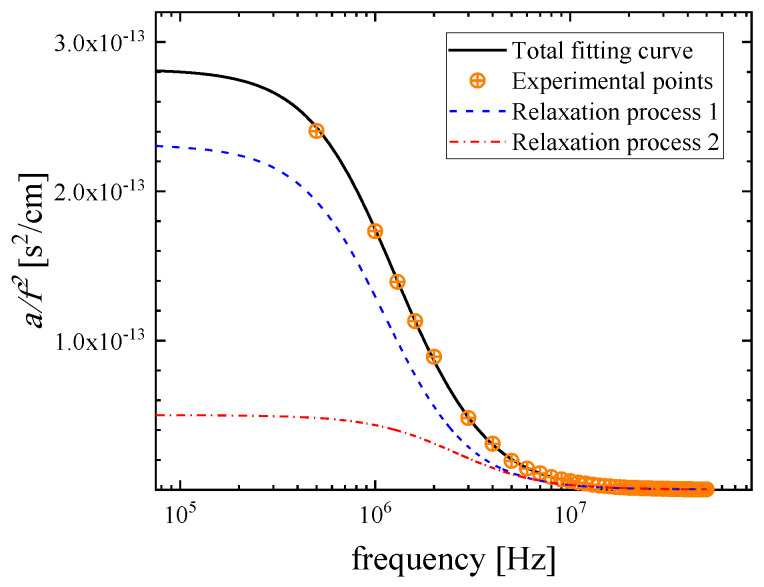
Fitting example of the ultrasound absorption for a solution corresponding to LiHMDS mole fraction of X_LiHMDS_ = 0.46 at 20 °C. The continuous solid black line denotes the total fitting, while the two dashed and dashed–dotted relaxation profiles correspond to distinct relaxation processes. Symbols represent experimental data.

It is interesting to note that the observed relaxation curves cannot be modeled with a single relaxation Debye-type profile for the full concentration range. A single relaxation is detected in the low- and in the high-mole fractions of LiHMDS, while in the intermediate region, two distinct Debye functions are necessary to sufficiently fit the experimental data. Thus, the double relaxation spectra were processed by a non-linear least-squares algorithm, and a representative fitting example of the ultrasound absorption is presented in Figure 4 for a solution with X_LiHMDS_ = 0.46 at 20 °C. The continuous solid black line denotes the total fitting, while the dashed and the dashed–dotted relaxation profiles correspond to distinct relaxation processes. The experimental data are represented by symbols.

**Figure 5:**
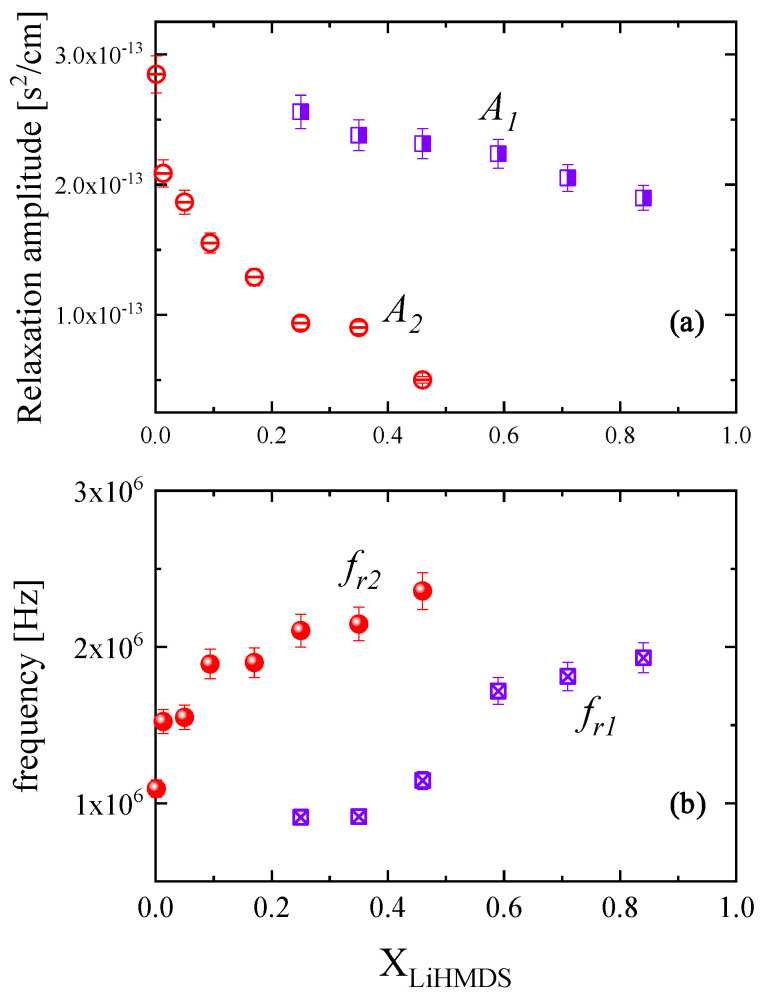
Mole fraction dependence of the relaxation amplitude *A* (**a**) and of the characteristic ultrasonic relaxation frequency *f_r_* (**b**) for the LiHMDS–toluene solutions at 20 °C.

Following the above fitting procedure, we estimated the free fitting parameters for all mole fractions, and the results are summarized in Table 2. The acoustic spectra corresponding to the 0.924 and 1.000 mole fraction were not analyzed due to their low signal-to-noise ratio. The characteristic ultrasonic relaxation frequencies and amplitudes for both relaxation processes as a function of LiHMDS mole fraction are presented in Figure 5a,b, respectively. The relaxation amplitudes of both relaxation processes detected in the acoustic spectra exhibit a clear monotonous decreasing trend with mole fraction, while the corresponding relaxation frequencies show the exact opposite behavior. Furthermore, as shown in Table 2, the classical contribution to absorption coefficient *B* reveals a rather constant value with solution concentration, which is almost three orders of magnitude lower than the relaxation amplitude of both relaxation mechanisms. The low-frequency relaxation mechanism observed in Figure 4 is assigned to the formation of trimers from monomer and dimer units (Equation (1)), while its high-frequency counterpart is attributed to the formation of the toluene-complexed LiHMDS dimer illustrated in Figure 1b (Equation (2)). The fitting results presented in Figure 5 further support our proposed assignments of the ultrasonic relaxation processes to these structural mechanisms.

Ultrasound absorption is sensitive to changes in particle size and molecular interaction; while sound velocity is sensitive to molecular changes, it does not remain constant in response to changes produced by pressure and depends on the degree of order of the molecules. The monotonous increase in the sound speed with increasing LiHMDS mole fraction, shown in Table 2, is indicative in the formation of higher-order aggregates in the overall structure. 

### 2.3. Kinetic Models of the Relaxation Processes 

It seems that bonding between solute molecules and/or solute and solvent molecules is the driving force behind the observed relaxations in the acoustic spectra. The formation and braking of the bonding between molecules induce perturbation in the acoustic wave propagation, which is accompanied by a change in the total volume and consequently by compressional relaxation. 

Considering Equation (1) which describes the formation of trimers from monomer and dimer units, we can define the relation between the relaxation frequency *f_r_*_,1_ and the LiHMDS concentration *C_LiHMDS_* as follows [11,12]:

(4) $$\frac{1}{\tau_{1}}=2\pi f_{r,1}=\left(k_{f,1}n^{2}\right){\left(C_{LiHMDS}\right)}^{n-1}+k_{b,1}$$

where $\tau_{1}$ is the characteristic relaxation time of the low-frequency process (process 1), while $k_{f,1}$ and $k_{b,1}$ are the forward and backward rate constants, respectively. Equation (4) describes the general form of the self-aggregation mechanism, and *n* is the corresponding aggregation number. 

**Figure 6:**
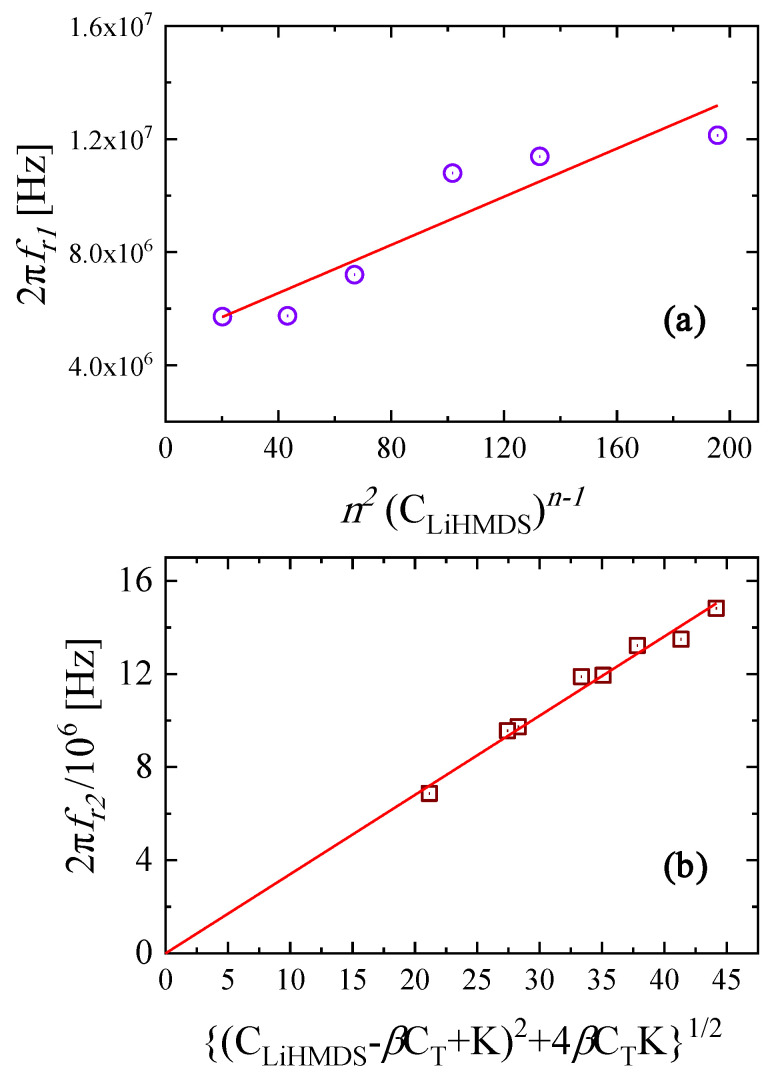
The plots of (**a**) $2\pi f_{r1}$ versus $n^{2}{\left(C_{LiHMDS}\right)}^{n-1}$ with *n* = 3 and (**b**) $2\pi f_{r2}/10^{6}$ versus ${\left\{{\left(C_{LiHMDS}-\beta C_{T}+K\right)}^{2}+4\beta C_{T}K\right\}}^{1/2}$ for the LiHMDS solutions at 20 °C. Lines correspond to linear fits with Pearson’s *r* equal to 0.93243 and 0.99956, respectively.

To determine the appropriate value of the aggregation number, several values of *n* were tested aiming to obtain the best linear fitting that corresponds to the least statistical error in the $2\pi f_{r,1}$ versus $n^{2}{\left(C_{LiHMDS}\right)}^{n-1}$ plot. The value *n* = 3 provided the best linear correlation with Pearson’s *r* = 0.93243. The results are shown in Figure 6a. Continuously increasing errors were received for aggregation number values higher than 3. From the linear dependency illustrated in Figure 6a, the forward and backward rate constants were evaluated from the slope and the intercept, respectively. These constants were found to be equal to $k_{f,1}=4.26\times 10^{4}{\;M}^{-1}s^{-1}$ and $k_{b,1}=4.85\times 10^{6}{\;s}^{-1}$. The equilibrium constant was estimated as follows:

(5) $$K_{1}=\frac{k_{f,1}}{k_{b,1}}=8.79\times 10^{3}\;M^{-1}$$

Monomeric species were not detected in non-polar solvents since they are less thermodynamically favorable [4,5,6].

The formation of the toluene-complexed LiHMDS dimer presented in Equation (2) is associated with the excess sound absorption observed in the high-frequency range, and its kinetics can be described by the following [13,14]:

(6) $$2\pi f_{r,2}=k_{b,2}\sqrt{{\left(C_{LiHMDS}-\beta C_{T}+K_{2}\right)}^{2}+4\beta C_{T}K_{2}}$$

where $f_{r,2}$ is the characteristic relaxation frequency of the high-frequency process (process 2), while $k_{b,2}$ is the backward rate constant, *K*_2_ is the equilibrium constant and *C_T_* is the concentration of toluene. Dimensionless parameter *β* describes the fraction of the unbounded toluene molecules. 

To determine the appropriate values of the equilibrium constant and parameter *β*, several test values of *K*_2_ and *β* were checked, aiming to obtain the best linear fitting that corresponded to the least statistical error in plot $2\pi f_{r,2}$ versus $\sqrt{{\left(C_{LiHMDS}-\beta C_{T}+K_{2}\right)}^{2}+4\beta C_{T}K_{2}}$. The results are shown in Figure 6b. The only restriction to the tested values was that *K*_2_ needed to be the same for all mole fractions of LiHMDS. The values of the equilibrium constant and the forward and backward rate constants that provided the best linear correlation with Pearson’s *r* = 0.999956 were found to be equal to $K_{2}=\frac{k_{f,2}}{k_{b,2}}=6.41\;M$, $k_{f,2}=4.10\times 10^{7}{\;s}^{-1}$ and $k_{b,2}=6.40\times 10^{6}{\;M}^{-1}s^{-1}$. The values of the dimensionless parameter *β* for all LiHMDS mole fractions are shown in Table 3.

The adiabatic compressibility of a fluid provides a measure of its compressibility, which is directly reflected in the dynamics of the system. This physical property can be determined from the sound velocity and mass density through the following equation:

(7) $$\kappa_{s}=\frac{1}{\rho u^{2}}$$

**Figure 7:**
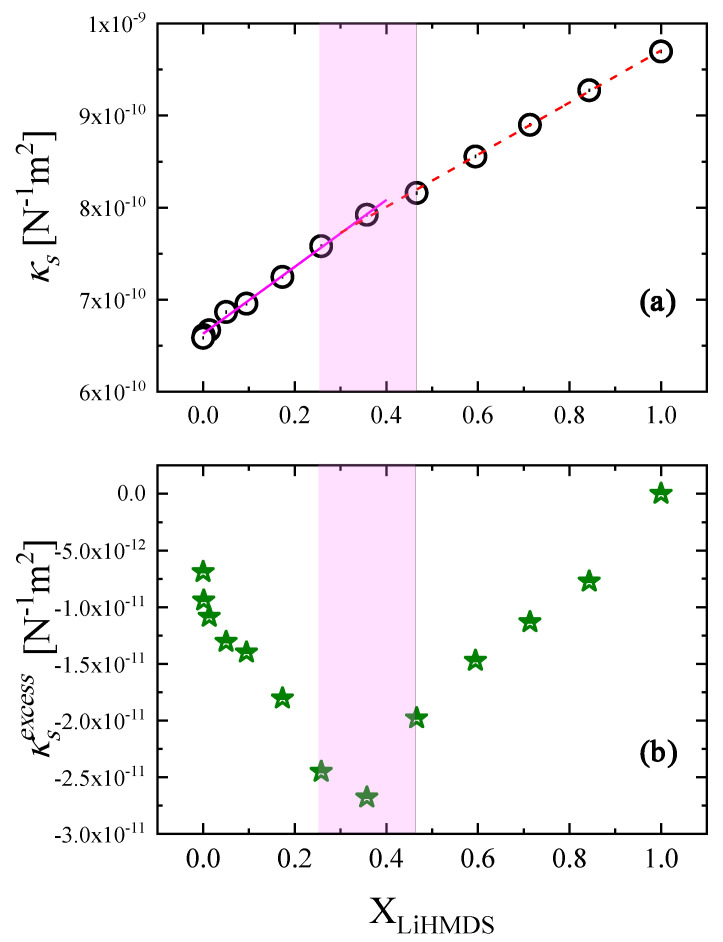
Concentration dependence of the adiabatic compressibility (**a**) and of the excess adiabatic compressibility (**b**) for all LiHMDS solutions studied at 20 °C. Linear fittings and dashed areas indicate the transition region between the two distinct structural mechanisms. See text for more details.

The experimental values of the isentropic compressibility are illustrated in Figure 7a as a function of LiHMDS mole fraction. Despite the monotonous increase in $\kappa_{s}$ with solution concentration, it seems that a sudden change near X_LiHMDS_ = 0.358 is observed. Furthermore, the trend below and above this crossover is linear. The structural and thermodynamic modifications that are caused by the mixing of the two liquids influence the ultrasonic wave propagation in the solutions, which in turn may provide valuable information on the intermolecular interactions. In the quantification of these alterations, it is beneficial to use the excess compressibility rather than the simple isentropic compressibility calculated by means of Equation (7). The excess isentropic compressibility is given by the following:

(8) $$\kappa_{S\left(excess\right)}=\kappa_{S\left(ideal\right)}-\kappa_{S\left(experimental\right)}$$

where the ideal property is estimated as follows:

(9) $$\kappa_{S\left(ideal\right)}=X\kappa_{S}^{0}\left(solute\right)+\left(1-X\right)\kappa_{S}^{0}\left(solvent\right)$$

The as-obtained results are presented in Figure 7b. A simple comparison between the experimental and the excess isentropic compressibility reveals that the change in the excess property is more pronounced and allows better surveillance of the behavior as a function of the LiHMDS mole fraction. From Figure 7b, the excess isentropic compressibility experiences a monotonous decrease up to X_LiHMDS_ = 0.358, while above this mole fraction, it experiences the exact opposite trend that is a clear monotonic increase. This behavior is analogous to that observed for experimental isentropic compressibility and indicates the interplay between the two structural mechanisms described by Equations (1) and (2) that coexist in the 0.258–0.466 mole fraction range. 

**Figure 8:**
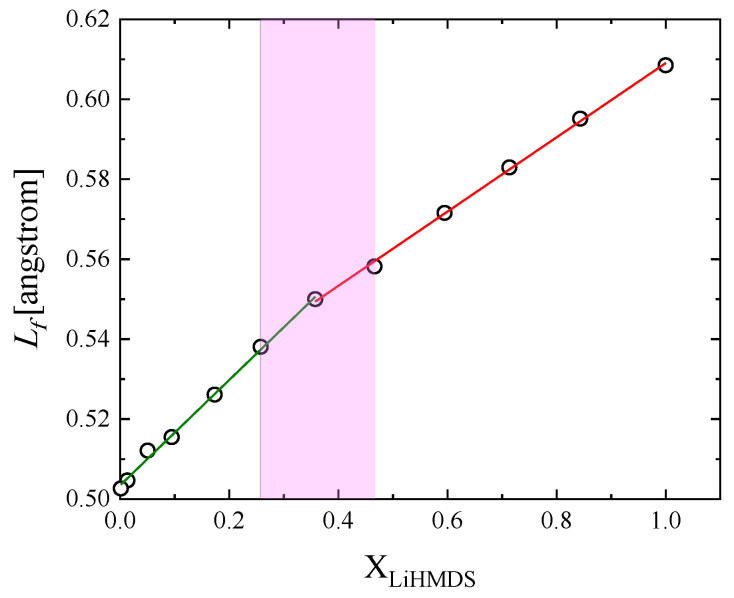
Intermolecular free length $L_{f}$ as a function of LiHMDS mole fraction at 20 °C.

Another interesting parameter is the intermolecular free length, which can be evaluated through the following empirical equation [15,16]:

(10) $$L_{f}=K\sqrt{\kappa_{s}}$$

with parameter K denoting the Jacobson’s constant. The calculated values for all mole fractions are presented in Figure 8. The intermolecular free length increases linearly with the LiHMDS mole fraction, although with different slopes below and above X_LiHMDS_ = 0.358, similar to the experimental adiabatic compressibility (Figure 7a). An increase in solution concentration results in a structure where LiHMDS molecules are closer to each other. Subsequently, an increase in the distance between toluene molecules and LiHMDS species is expected, which is verified experimentally in Figure 8.

For the *i*-th (*i* = 1, 2) structural mechanisms described by Equations (1) and (2), the isentropic standard volume change $\Delta V_{s}$ can be defined as follows [17]:

(11) $$\Delta V_{s,i}=\sqrt{\frac{ARTf_{r,i}}{\pi \rho u\Gamma_{i}}}$$

where ρ is the mass density of the solution, *T* is the absolute temperature, *u* is the speed of sound in the solution and *R* is the gas constant. Γ*_i_* is the concentration parameter, while *A_i_* and *f_r,i_* correspond to the relaxation amplitude and frequency for each mechanism, respectively. 

In general, the concentration parameter Γ reflects the progress of a specific reaction and, for the two relaxation mechanisms occurring in our case, can be estimated as the following [18]:

(12) $$\Gamma_{1}=\frac{9}{{\left[LiHMDS\right]}_{3}}+\frac{4}{{\left[LiHMDS\right]}_{2}}+\frac{1}{\left[LiHMDS\right]}$$

and

(13) $$\Gamma_{2}=\frac{1}{{\left[LiHMDS\right]}_{2}}+\frac{1}{\left[Toluene\right]}+\frac{1}{Toluene\cdot {\left[LiHMDS\right]}_{2}}$$

**Figure 9:**
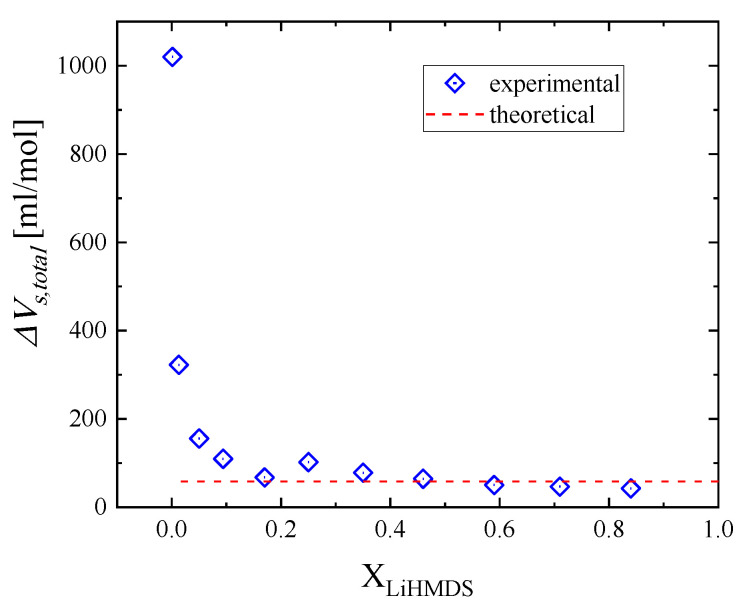
Sum of the isentropic volume changes as a function of mole fraction due to LiHMDS trimer formation (relaxation process 1) and to the mixed aggregate LiHMDS dimer–toluene (relaxation process 2). Symbols represent the experimental values, while the dotted line denotes the theoretically predicted volume change, respectively.

The as-obtained values of the isentropic volume changes attributed to the LiHMDS trimer formation (relaxation process 1) and to the mixed aggregate LiHMDS dimer–toluene (relaxation process 2) are summarized in Table 4. The variation in the total volume as a function of mole fractions is presented in Figure 9. The results reveal a decreasing trend, which is stronger at lower mole fractions and levels off to the theoretically predicted volume changes for higher mole fractions. 

### 2.4. Thermodynamic Analysis of the Relaxation Processes

**Figure 10:**
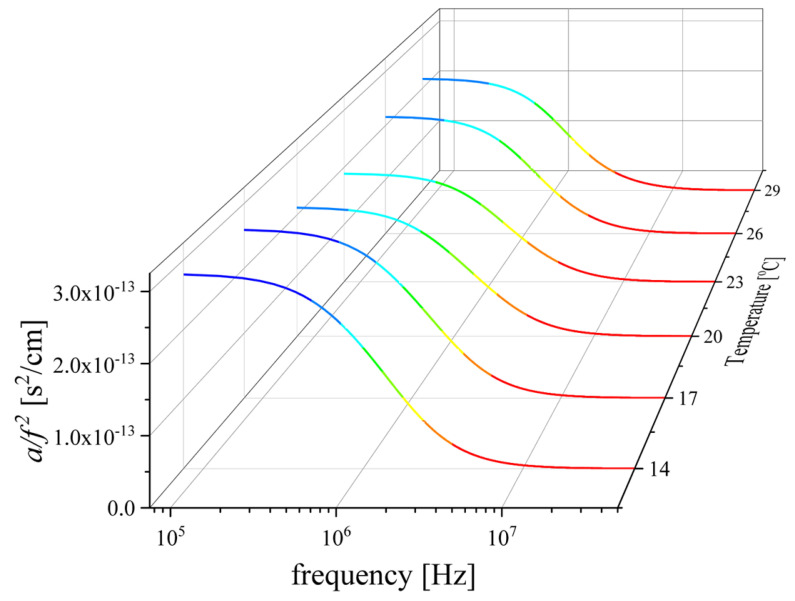
Ultrasound absorption spectra $\left(a/f^{2}\right)$ as a function of frequency for all temperatures studied. Measurements were performed for the LiHMDS–toluene solution, which correspond to the 0.466 mole fraction of LiHMDS. Lines represent the total fitting relaxation profiles for each temperature.

**Figure 11:**
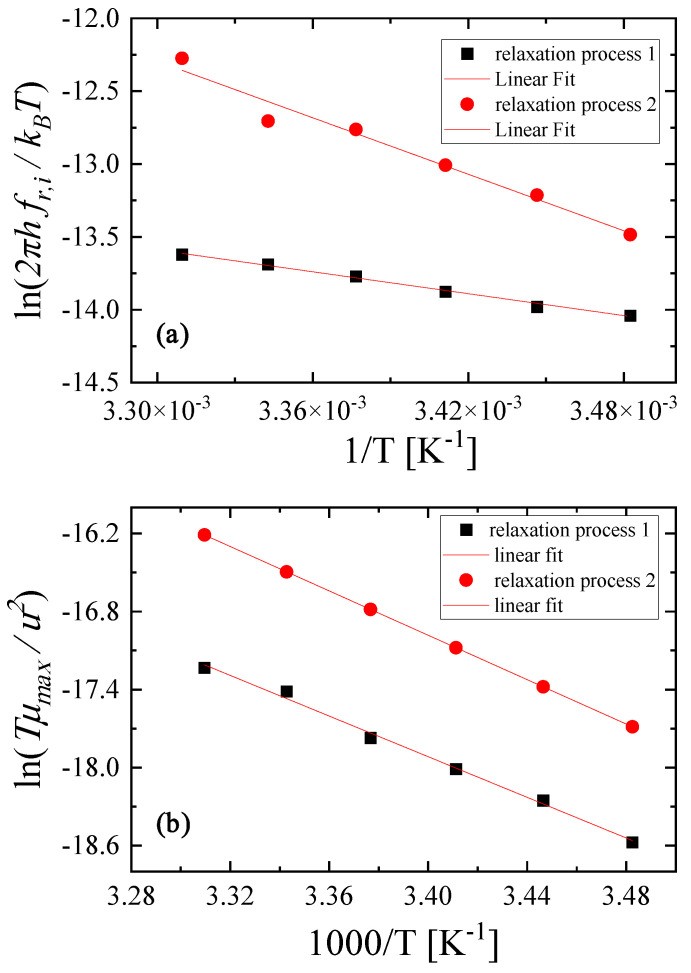
Plots of ln2πhfr,ikΒΤ versus 1/T (**a**) and of lnTμmaxu2 versus 1000/T (**b**) for relaxation processes 1 and 2, respectively. See text for more details.

In Figure 10, the sound absorption coefficients as a function of frequency for all temperatures are shown. The solution studied corresponds to a 0.466 mole fraction of LiHMDS. All spectra were fitted with a double Debye–type function. In the context of Eyring’s theory, the characteristic frequency for each relaxation mechanism follows a temperature dependency, which is given by the following [19,20,21]:

(14) $$\frac{f_{r,i}}{T}=\frac{k_{B}}{2\pi h}\exp\left(-\frac{\Delta H_{i}^{*}}{RT}\right)\exp\left(\frac{\Delta S_{i}^{*}}{R}\right)$$

where $k_{B}$ and h are Boltzmann’s and Planck’s constants, respectively. $\Delta H_{i}^{*}$ represents the activation enthalpy and $\Delta S_{i}^{*}$ denotes the activation entropy. By plotting $ln\left(\frac{2\pi hf_{r,i}}{k_{B}T}\right)$ as a function of the reciprocal temperature, one can evaluate the activation enthalpy directly from the slope of the diagram and the activation entropy from the corresponding intercept. The trend is expected to be linear and indeed, as shown in Figure 11a, the data in the Arrhenius-type diagram seem to follow a linear dependency. 

The activation enthalpies were calculated to be equal to $\Delta H_{1}^{*}=4.98\pm 0.32\;\mathrm{kcal}/\mathrm{mol}$ and $\Delta H_{2}^{*}=12.84\pm 1.12\;\mathrm{kcal}/\mathrm{mol}$ for relaxation process 1 and 2, respectively. The entropy changes were found to be equal to $\Delta S_{1}^{*}=10.57\pm 1.09\;\mathrm{cal}/\mathrm{molK}$ and $\Delta S_{2}^{*}=17.94\pm 3.79\;\mathrm{cal}/\mathrm{molK}$, revealing only a minor contribution to the free energy change. 

For the *i*-th (*i* = 1, 2) structural mechanisms defined by Equations (1) and (2), the amplitude of the relaxation is given by the following [18]:

(15) $$\mu_{max,i}=\frac{1}{2}A_{i}uf_{r,i}$$

Furthermore, the temperature dependency of the relaxation amplitude is mainly defined by the exponential factor exp−ΔHi0RT, and it was found that the following equation holds [21]:

(16) $$\frac{T\mu_{max,i}}{u^{2}}=\left(constant\right)\times exp\left(-\frac{\Delta H_{i}^{0}}{RT}\right)$$

Thus, from the Arrhenius-type plot of $\ln\left(\frac{T\mu_{max,i}}{u^{2}}\right)$ versus  1000T presented in Figure 11b, we can determine the difference in enthalpy directly from the slope. The as-obtained values of enthalpy differences were equal to $\Delta H_{1}^{0}=15.96\pm 2.88\;\mathrm{kcal}/\mathrm{mol}$ and $\Delta H_{2}^{0}=16.93\pm 0.17\;\mathrm{kcal}/\mathrm{mol}$ for relaxation process 1 and 2, respectively. The linear trend observed in Figure 11b reveals that the enthalpy differences $\Delta H_{i}^{0}$ were temperature-independent, at least in the temperature range studied here. 

## 3. Materials and Methods

### 3.1. Solutions

For the preparation of the solutions, Lithium bis(trimethylsilyl)amide (97%, Sigma-Aldrich, Burlington, MA, USA) and toluene (99.5%, Fluka, Charlotte, North Carolina, USA) were used without any further purification. The binary solutions cover a wide range of concentrations. The mole fractions and the corresponding concentrations are presented in Table 5. Only fresh solutions were used for the complete set of measurements. The reaction of water with LiHMDS is known to be violent [22]. In the present work, we did not use water as a solvent, and caution was paid to avoid any contact of LiHMDS with moisture.

### 3.2. Ultrasonic Relaxation Spectroscopic Measurements

The sound absorption coefficient was measured by means of the parallel-path pulse method as a function of concentration and temperature with an experimental error less than ±5% [22]. A set of two wide-band piezoelectric elements was utilized as the transmitter and receiver of the ultrasonic wave, respectively. The liquid sample was placed into a temperature-controlled cylindrical acoustic cell, and the two piezoelectric elements were attached to the opposite faces of the cell. Temperature was controlled within ±0.01 °C. A common ultrasonic medical gel was applied between the cell and the transducers to achieve better contact and thus the maximum sound transmission. Utilizing the same experimental setup, ultrasound velocity measurements were performed via the pulse-echo overlap technique with an experimental error less than ±0.01% [23]. 

The mass densities of all solutions were measured with a temperature-controlled density-meter (DM 60, Anton Paar, Germany GmbH, Scharnhausen, Ostfildern, Germany) with an accuracy of ±0.0001 g/cm^3^. More details concerning the setup and the experimental protocols can be found elsewhere [24]. 

### 3.3. Theoretical Calculations

The structures of LiHMDS and toluene were fetched as SDF digital files from the PubChem electronic database and were optimized by employing Density Functional Theory (B3LYP) methodology combined with the 6-311G(d,p) basis set and tight convergence criteria. The theoretical entropy and the corresponding vibrational properties of the LiHMDS-monomer, LiHMDS-dimer and LiHMDS-trimer complex with one toluene molecule were calculated after optimization. Molecular volumes were estimated theoretically as the volume inside a contour of a particular electron density. All calculations were carried out in vacuum with the use of the Gaussian 09 program [25]. 

Molecular docking calculations were conducted with the AutoDock software (version 4.2) to investigate the interaction between toluene and LiHMDS species. The calculation was performed with the optimized structures. 

The LiHMDS trimer was kept stationary at the center of the simulation box (receptor), while the toluene molecule was left to move freely inside the boundaries of the simulation box (ligand). The dimensions of the box were set at 25 Å × 25 Å × 25 Å, and the grid spacing was fixed at a default value of 0.375 Å. The rotatable number of bonds for toluene was set at maximum. The charges were assigned with the help of Gasteiger charges. The best poses were selected through the Lamarckian genetic algorithm (LGA) [26]. The final most plausible pose after molecular docking calculation was also optimized with the same basis set. 

## 4. Conclusions

The combination of ultrasonic relaxation spectroscopy with computational molecular docking and DFT methods allowed us to evaluate how solvent coordination modulates the dynamical processes detected in the acoustic spectra. A wide-concentration and temperature-dependent study was undertaken by means of the pulse-echo technique.

Molecular docking calculations revealed the formation of trimers from monomer and dimer units, as well as the formation of an aggregate between toluene and LiHMDS dimer species. DFT methodologies were used to evidence the stability of all species involved. The concentration dependence of the acoustic spectra revealed the presence of two discrete relaxation processes. Low-frequency relaxation dominated in the high-mole fractions of LiHMDS and was attributed to the formation of trimer species. On the other hand, high-frequency relaxation was the main process in the low-mole fraction of LiHMDS and was related with the formation of the hetero-aggregate between toluene and LiHMDS dimer species. 

From the concentration dependence of the relaxation frequencies for both relaxation mechanisms, the relevant kinetic properties were obtained. The forward and backward rate constants for the two processes were determined to be equal to $k_{f,1}=4.26\times 10^{4}{\;M}^{-1}s^{-1}$, $k_{b,1}=4.85\times 10^{6}{\;s}^{-1}$ and $k_{f,2}=4.10\times 10^{7}{\;s}^{-1}$, $k_{b,2}=6.40\times 10^{6}{\;M}^{-1}s^{-1}$, respectively. From the forward and backward rate constants, the equilibrium constants were computed as $K_{1}=8.79\times 10^{3}\;M^{-1}$ $K_{2}=6.41\;M$ for the self- and hetero-aggregation reaction.

From the temperature dependence of the acoustic spectra, the thermodynamic characteristics of both structural mechanisms were evaluated. The activation enthalpy and entropy were estimated to be equal to $\Delta H_{1}^{*}=4.98\pm 0.32\;\mathrm{kcal}/\mathrm{mol}$ and $\Delta S_{1}^{*}=10.57\pm 1.09\;\mathrm{cal}/\mathrm{molK}$ for the self-aggregation of LiHMDS, while for the hetero-aggregation mechanism, the corresponding thermodynamic parameters were found to be $\Delta H_{2}^{*}=12.84\pm 1.12\;\mathrm{kcal}/\mathrm{mol}$ and $\Delta S_{2}^{*}=17.94\pm 3.79\;\mathrm{cal}/\mathrm{molK}$, respectively. The enthalpy differences for both processes were also calculated from the temperature dependence of the acoustic parameters and found to be equal to $\Delta H_{1}^{0}=15.96\pm 2.88\;\mathrm{kcal}/\mathrm{mol}$ and $\Delta H_{2}^{0}=16.93\pm 0.17\;\mathrm{kcal}/\mathrm{mol}$ for relaxation process 1 and 2, respectively. 

The total volume changes associated with the above structural mechanisms were also evaluated both experimentally and theoretically and were found to be comparable despite the fact that the theoretical calculation was performed in vacuum without taking into consideration any intermolecular interactions. 

All of the above outcomes are practically ascribed to variations in interactions at the molecular level that took place in the structure of LiHMDS–toluene solutions.

## Figures and Tables

**Table 1:** Theoretically predicted molecular volumes.

Species	Molekular Volume (cm^3^/mol)
LiHMDS monomer	154.312
LiHMDS dimer	296.290
LiHMDS trimer	496.820
Toluene	86.081
Toluene-complexed LiHMDS dimer	502.320

**Table 2:** Concentration, mole fraction and sound speed values for the LiHMDS–toluene solutions.

*X_LiHMDS_*	*f*_*r*1_ (×10^6^ Hz)	*A*_1_ (×10^−13^ s^2^/cm)	*f*_*r*2_ (×10^6^ Hz)	*A*_2_ (×10^−13^ s^2^/cm)	*B* (×10^−16^ s^2^/cm)	*u* (m/s)
1	-	-	-	-	-	1093.48
0.924	-	-	-	-	-	1095.84
0.843	1.93	1.90	-	-	2.75	1102.8
0.714	1.81	2.05	-	-	2.64	1126.5
0.595	1.72	2.24	-	-	2.63	1149.4
0.466	1.15	2.32	2.36	0.50	2.01	1177.9
0.358	0.92	2.38	2.15	0.90	2.19	1205.2
0.258	0.91	2.56	2.10	0.94	2.36	1233.1
0.173	-	-	1.90	1.29	1.83	1260.2
0.094	-	-	1.89	1.55	1.87	1286.9
0.050	-	-	1.55	1.86	1.38	1295.3
0.013	-	-	1.52	2.09	1.37	1315.8
0.001	-	-	1.09	2.85	1.44	1321.1

**Table 3:** Concentration dependency of parameter *β*.

X_LiHMDS_	C_LiHMDS_ (M)	β
1	5.14	-
0.924	4.92	-
0.843	4.66	-
0.714	3.84	-
0.595	3.36	-
0.466	2.73	0.20
0.358	2.19	0.24
0.258	1.50	0.28
0.173	1.17	0.20
0.094	0.58	0.25
0.050	0.31	0.15
0.013	0.12	0.10
0.001	0.01	0.01

**Table 4:** Isentropic volume changes due to LiHMDS trimer formation (relaxation process 1) and to mixed aggregate LiHMDS dimer–toluene (relaxation process 2).

*X*_*LiHMDS*_	Δ*V*_*s*1_ (mL/mol)	Δ*V*_*s*2_ (mL/mol)	Δ*V*_*s,total*_ (mL/mol)
1	--	--	--
0.924	--	--	--
0.843	42.6	0	42.6
0.714	46.8	0	46.8
0.595	50.3	0	50.3
0.466	45.9	18.6	64.5
0.358	46.2	31.8	78.1
0.258	57.2	44.8	102.1
0.173	0	67.6	67.6
0.094	0	109.5	109.5
0.050	0	155.6	155.6
0.013	0	322.6	322.6
0.001	0	1020.0	1020.0
theoretical	46.2	12.1	58.3

**Table 5:** Concentrations and mole fractions of the LiHMDS–toluene solutions.

Solution	X_LiHMDS_	C_LiHMDS_ (M)	C_toluene_ (M)
1	1	5.14	0
2	0.924	4.92	0.41
3	0.843	4.66	0.87
4	0.714	3.84	1.54
5	0.595	3.36	2.29
6	0.466	2.73	3.12
7	0.358	2.19	3.94
8	0.258	1.50	4.31
9	0.173	1.17	5.57
10	0.094	0.58	5.53
11	0.050	0.31	5.89
12	0.013	0.12	9.13
13	0.001	0.01	9.34

## Data Availability

Data are available upon request from the corresponding author.

## Supplementary Materials

The following supporting information can be downloaded at: https://www.mdpi.com/article/10.3390/molecules29040813/s1, Figure S1. LiHMDS–toluene complex. The figure depicts the angles between the N-Li-N (109.1°) and the angle of N-Li-η6-toluene (113.3°). Figure S2. The theoretical representation of LiHMDS–toluene complex with labeled atoms for easier identification (see Table S1). Table S1. The bond distances and angles of the LiHMDS–toluene complex.

## References

[1] Danheiser R.L., Miller R.F., Brisbois R.G. (1996). Detrifluoroacetylative diazo group transfer: (E)-1-diazo-4-phenyl-3-buten-2-one. Org. Synth..

[2] Wu G., Huang M. (2006). Organolithium Reagents in Pharmaceutical Asymmetric Processes. Chem. Rev..

[3] Lappert M., Protchenko A., Power P., Seeber A. (2009). Metal Amide Chemistry.

[4] Lucht B.L., Collum D.B. (1995). Ethereal Solvation of Lithium Hexamethyldisilazide: Unexpected Relationships of Solvation Number, Solvation Energy, and Aggregation State. J. Am. Chem. Soc..

[5] Lucht B.L., Collum D.B. (1996). Lithium Ion Solvation: Amine and Unsaturated Hydrocarbon Solvates of Lithium Hexamethyldisilazide (LiHMDS). J. Am. Chem. Soc..

[6] Neufeld R., Michel R., Herbst-Irmer R., Schçne R., Stalke D. (2016). Introducing a Hydrogen-Bond Donor into a Weakly Nucleophilic Brønsted Base: Alkali Metal Hexamethyldisilazides (MHMDS, M= Li, Na, K, Rb and Cs) with Ammonia. Chem. Eur. J..

[7] Rogers R.D., Atwood J.L., Grüning R. (1978). The crystal structure of N-lithiohexamethyldisilazane, [LiN(SiMe_3_)_2_]_3_. J. Organomet. Chem..

[8] Popenova S., Mawhinney R.C., Schreckenbach G. (2007). Density Functional Study of Lithium Hexamethyldisilazide (LiHMDS) Complexes: Effects of Solvation and Aggregation. Inorg. Chem..

[9] Slutsky L.J., Edmonds P.D. (1981). Ultrasonic Chemical Relaxation Spectroscopy.

[10] Herzfeld K.F., Litovitz T.A. (1959). Absorption and Dispersion of Ultrasonic Waves.

[11] Nishikawa S., Kamimura E. (2011). Dynamic characteristic of amitriptyline in water by ultrasonic relaxation method and molecular orbital calculation. J. Phys. Chem. A.

[12] Nishikawa S., Haraguchi H., Fukuyama Y. (1991). Effect of ether oxygen on proton transfer and aggregation reactions of amines in water by ultrasonic absorption method. Bull. Chem. Soc. Jpn..

[13] Nishikawa S., Mashima M., Yasunaga T. (1975). Ultrasonic Absorption Mechanism in an Aqueous Solution of n-Propyl Alcohol. Bull. Chem. Soc. Jpn..

[14] Nishikawa S., Kuramoto N., Uchiyama T. (1994). Ultrasonic Relaxation Associated with Solute-Solvent Interaction in an Aqueous Solution of 5-Methoxy-1-pentanol. Bull. Chem. Soc. Jpn.

[15] Jacobson B. (1952). Ultrasonic Velocity in Liquids and Liquid Mixtures. J. Chem. Phys..

[16] Jacobson B. (1952). Intermolecular Free Lengths in Liquids in Relation to Compressibility, Surface Tension and Viscosity. Acta Chem. Scand..

[17] Tsigoias S., Papanikolaou M.G., Kabanos T.A., Kalampounias A.G. (2021). Structure and dynamics of aqueous norspermidine solutions: An in situ ultrasonic relaxation spectroscopic study. J. Phys. Condens. Matter..

[18] Kaatze U., Hushcha T.O., Eggers F. (2000). Ultrasonic Broadband Spectrometry of Liquids: A Research Tool in Pure and Applied Chemistry and Chemical Physics. J. Solution Chem..

[19] Blandamer M.J. (1973). Introduction to Chemical Ultrasonics.

[20] Ensminger D., Bond L.J. (2011). Ultrasonics: Fundamentals, Technologies, and Applications.

[21] Stogiannidis G., Tsigoias S., Kalampounias A.G. (2020). Conformational energy barriers in methyl acetate–Ethanol solutions: A temperature-dependent ultrasonic relaxation study and molecular orbital calculations. J. Mol. Liq..

[22] Jäger S., Meyer P., Feichtner K.-S., Henkel S., Schwaab G.W., Gessner V.H., Havenith M. (2022). Reaction of lithium hexamethyldisilazide (LiHMDS) with water at ultracold conditions. Phys. Chem. Chem. Phys..

[23] Kouderis C., Siafarika P., Kalampounias A.G. (2021). Molecular relaxation dynamics and self-association of dexamethasone sodium phosphate solutions. Chem. Pap..

[24] Kouderis C., Siafarika P., Kalampounias A.G. (2021). Disentangling proton-transfer and segmental motion relaxations in poly-vinyl-alcohol aqueous solutions by means of ultrasonic relaxation spectroscopy. Polymer.

[25] Frisch M.J., Trucks G.W., Schlegel H.B., Scuseria G.E., Robb M.A., Cheeseman J.R., Scalmani G., Barone V., Petersson G.A., Nakatsuji H. (2009). Gaussian 09, Revision A.02.

[26] Kouderis C., Tsigoias S., Siafarika P., Kalampounias A.G. (2023). The Effect of Alkali Iodide Salts in the Inclusion Process of Phenolphthalein in β-Cyclodextrin: A Spectroscopic and Theoretical Study. Molecules.

