# Supplementary material for: The Identification of Structural Changes in the Lithium Hexamethyldisilazide–Toluene System via Ultrasonic Relaxation Spectroscopy and Theoretical Calculations
Source: Molecules. 2024 Feb 9;29(4):813. doi: 10.3390/molecules29040813 (PMC10892886; doi:10.3390/molecules29040813)
Supplement: Supplementary file 1 [file molecules-29-00813-s001.zip › molecules-2842456-supplementary.pdf]

Supplementary Information

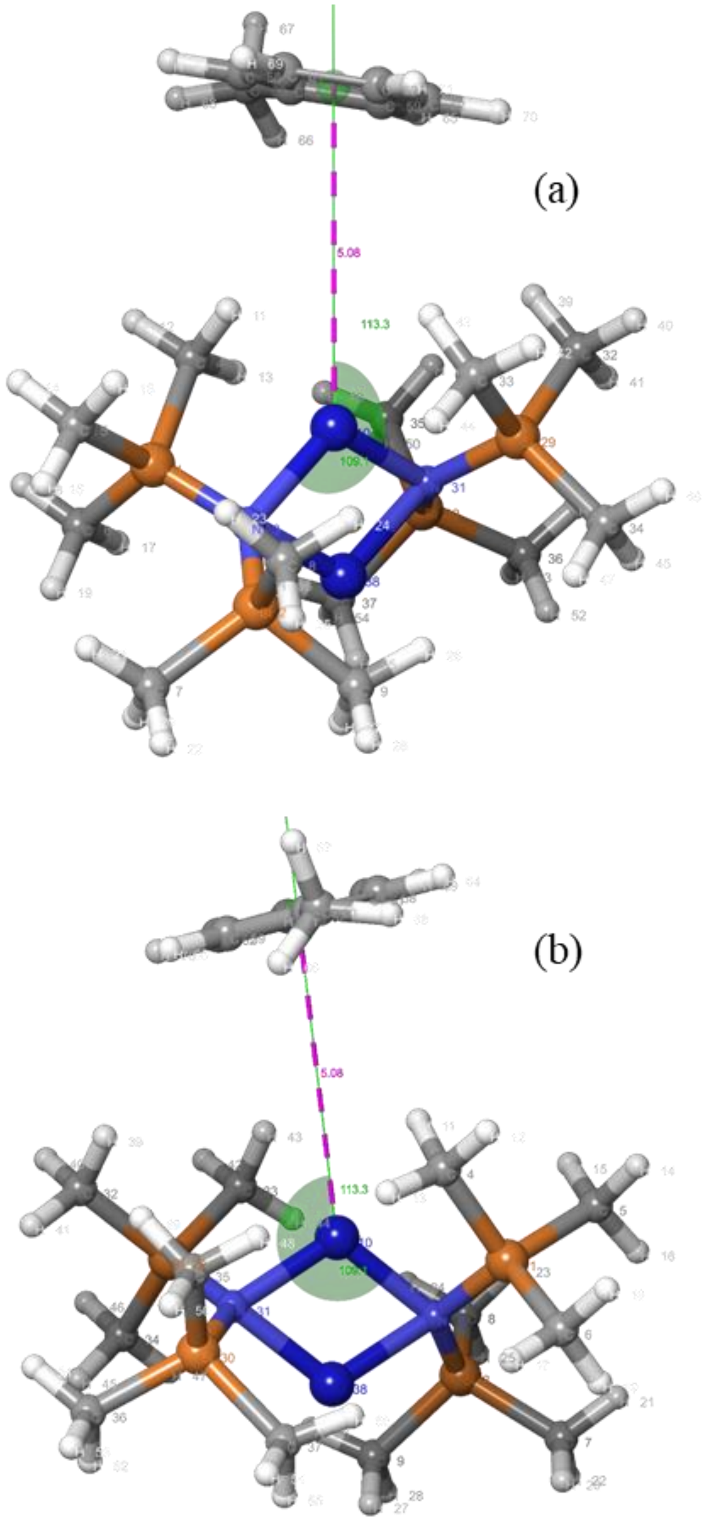

Figure S1. LiHMDS-toluene complex. The figure depicts the angles between the N-Li-N (109.1^0) and the angle of N-Li-η^6-toluene (113.3^o).

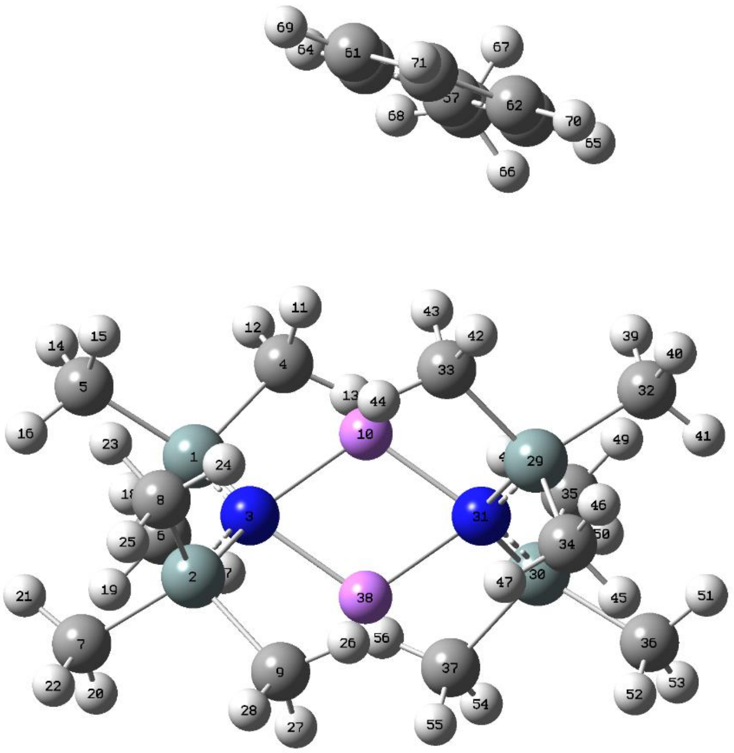

Figure S2. The theoretical representation of LiHMDS-toluene complex with labeled atoms for easier identification (see **Table S1)**.

Table S1. The bond distances and angles of the LiHMDS-toluene complex.

Tag	Symbol	NA	Bond (Å)	Angle (°)
1	Si			
2	Si	1	3.070	
3	N	2	1.723	27.047
4	C	1	1.915	107.074
5	C	1	1.896	114.968
6	C	1	1.895	114.221
7	C	2	1.896	92.992
8	C	2	1.894	110.527
9	C	2	1.918	130.679
10	Li	3	1.991	131.051
11	H	4	1.099	113.605
12	H	4	1.094	109.798
13	H	4	1.097	113.498
14	H	5	1.095	110.830
15	H	5	1.094	111.320
16	H	5	1.093	112.123
17	H	6	1.094	111.586
18	H	6	1.095	111.597
19	H	6	1.094	110.945

20	H	7	1.094	111.442
21	H	7	1.093	112.075
22	H	7	1.095	110.776
23	H	8	1.094	110.779
24	H	8	1.094	111.691
25	H	8	1.095	111.620
26	H	9	1.097	113.701
27	H	9	1.099	112.876
28	H	9	1.093	109.992
29	Si	10	2.765	128.725
30	Si	29	3.075	70.004
31	N	30	1.722	26.858
32	C	29	1.896	121.368
33	C	29	1.914	61.808
34	C	29	1.895	131.058
35	C	30	1.894	110.673
36	C	30	1.896	93.039
37	C	30	1.917	130.397
38	Li	31	1.980	95.759
39	H	32	1.094	111.008
40	H	32	1.095	110.916
41	H	32	1.093	112.178
42	H	33	1.094	109.953
43	H	33	1.099	112.973
44	H	33	1.096	113.438
45	H	34	1.094	110.986
46	H	34	1.095	111.524
47	H	34	1.094	111.648
48	H	35	1.094	111.629
49	H	35	1.094	110.730
50	H	35	1.095	111.683
51	H	36	1.093	112.112
52	H	36	1.094	111.446
53	H	36	1.095	110.743
54	H	37	1.093	110.146
55	H	37	1.099	112.705
56	H	37	1.097	113.648
57	C	4	4.429	160.902
58	C	57	1.398	77.402
59	C	57	1.399	100.101
60	C	57	1.509	93.471
61	C	58	1.394	121.050
62	C	59	1.392	121.038
63	C	61	1.393	120.173
64	H	58	1.085	119.370
65	H	59	1.085	119.391
66	H	60	1.092	111.307
67	H	60	1.095	111.012
68	H	60	1.091	111.413
69	H	61	1.084	119.750

70	H	62	1.084	119.751
71	H	63	1.083	120.322
